# Applications for detection of acute kidney injury using electronic medical records and clinical information systems: workgroup statements from the 15^th^ ADQI Consensus Conference

**DOI:** 10.1186/s40697-016-0100-2

**Published:** 2016-02-26

**Authors:** Matthew T. James, Charles E. Hobson, Michael Darmon, Sumit Mohan, Darren Hudson, Stuart L. Goldstein, Claudio Ronco, John A. Kellum, Sean M. Bagshaw, For the Acute Dialysis Quality Initiative (ADQI) Consensus Group

**Affiliations:** Departments of Medicine and Community Health Sciences, Cumming School of Medicine, University of Calgary, Calgary, Canada; Department of Health Services Research, Management and Policy, University of Florida, Gainesville, Florida; Department of Intensive Care Medicine, Saint-Etienne University Hospital, Saint-Priest-En-Jarez, France; Department of Medicine, Division of Nephrology, Columbia University Medical Center, New York, NY USA; Division of Critical Care Medicine, Faculty of Medicine and Dentistry, University of Alberta, Edmonton, Canada; Department of Nephrology, Dialysis and Transplantation, International Renal Research Institute of Vicenza, San Bortolo Hospital, Vicenza, Italy; Department of Pediatrics, Division of Pediatric Nephrology, Cincinnati Children’s Hospital Medical Center, Cincinnati, OH USA; Center for Critical Care Nephrology, Department of Critical Care Medicine, University of Pittsburgh, Pittsburgh, PA USA

**Keywords:** Acute kidney injury, Detection, Clinical informatics, Clinical decision support

## Abstract

Electronic medical records and clinical information systems are increasingly used in hospitals and can be leveraged to improve recognition and care for acute kidney injury. This Acute Dialysis Quality Initiative (ADQI) workgroup was convened to develop consensus around principles for the design of automated AKI detection systems to produce real-time AKI alerts using electronic systems. AKI alerts were recognized by the workgroup as an opportunity to prompt earlier clinical evaluation, further testing and ultimately intervention, rather than as a diagnostic label. Workgroup members agreed with designing AKI alert systems to align with the existing KDIGO classification system, but recommended future work to further refine the appropriateness of AKI alerts and to link these alerts to actionable recommendations for AKI care. The consensus statements developed in this review can be used as a roadmap for development of future electronic applications for automated detection and reporting of AKI.

**Electronic supplementary material:**

The online version of this article (doi:10.1186/s40697-016-0100-2) contains supplementary material, which is available to authorized users.

## Abrégé

Les dossiers médicaux électroniques et les systèmes de renseignements cliniques sont de plus en plus utilisés dans les hôpitaux. Ces éléments pourraient être mis à profit pour faciliter le dépistage de l’insuffisance rénale aigüe (IRA) et améliorer les soins offerts aux patients qui en souffrent. Lors de la dernière réunion du *Acute Dialysis Quality Initiative* (ADQI), un groupe de travail s’est réuni pour établir un consensus autour de principes régissant la constitution d’un système automatisé de détection de l’IRA. Un système qui permettrait de produire des alertes en temps réel pour dépister les cas d’IRA (alertes IRA). Le groupe de travail a reconnu que de telles alertes représenteraient des opportunités de procéder à une évaluation clinique ou un dépistage précoce de la maladie et donc, à des interventions plus rapides, plutôt que de ne constituer qu’un indicateur diagnostique. Les membres du groupe de travail se sont entendus pour que le système d’alertes IRA soit développé en se basant sur la classification établie par le KIDGO. Ils ont toutefois recommandé que des travaux ultérieurs soient effectués pour raffiner les alertes et pour que celles-ci soient suivies de recommandations applicables et assorties d’un plan concret de soins à offrir aux patients. Les déclarations consensuelles présentées dans ce compte-rendu pourraient constituer le plan de développement pour la mise au point d’applications électroniques permettant la détection et le signalement de cas d’IRA de façon automatisée.

## Background

Acute kidney injury (AKI) is a common complication in patients hospitalized for a range of medical conditions and surgical procedures. AKI usually occurs in susceptible patients following episodes of low blood pressure, volume depletion, sepsis, use of diagnostic imaging contrast media, and/or nephrotoxic drug exposure [1, 2]. The incidence of AKI has increased more than 4-fold over the last two decades and AKI is expected to continue to rise in frequency due to the growing prevalence of risk factors, accompanied by the expanding use of medications, diagnostic imaging, and surgical interventions that can lead to AKI [3–5]. AKI is usually accompanied by few specific symptoms or signs, which can delay recognition, but its progression can be avoided or reversed with early recognition [1, 4].

In 2012, Kidney Disease Improving Global Outcomes (KDIGO) published clinical practice guidelines for AKI prevention, identification, and treatment [6]. These guidelines include specific recommendations for; 1) identification of patients who are susceptible to AKI, 2) use of validated laboratory and urine output criteria for AKI identification and staging (Table 1), and 2) stage-based management approaches for AKI. However, implementing these recommendations in clinical practice remains challenging. Patients who develop AKI are cared for by diverse providers from several different medical and surgical disciplines, many of whom may not be aware of guideline recommendations [7, 8]. Lack of recognition of AKI by care providers leads to delayed intervention and has been identified as a barrier to optimal care [7].

**Table 1:** The KDIGO staging system for AKI

AKI Stage	Serum creatinine criteria	Urine output criteria
1	Increase > 26.4 μmol/L	<0.5 mL · Kg^−1^ · h^−1^ for 6 to 12 h
	*or* 1.5-1.9 times baseline	
2	Increase 2.0 -2.9 times baseline	<0.5 mL · Kg^−1^ · h^−1^ for more than 12 h
3	Increase creatinine > 354 μmol/L	<0.3 mL · Kg^−1^ · h^−1^ for 24 h
	*or* 3 times baseline	
		or anuria for 12 h
	*or* initiation of RRT

**Table 2:** Features that may influence the performance of automated AKI alerts based on the KDIGO AKI criteria

KDIGO AKI criteria	Feature
Serum creatinine	Calibration of measure according to IDMS standard
	Optimal measurement using enzymatic assay [15]
	Comparison across laboratories or measurement techniques [16]
	Relevancy of e-alert systems using estimated baseline creatinine
	If previous creatinine available, chosen definition of baseline creatinine
	Management of outliers measures
	Significance of small changes in serum creatinine in patients with low weight/body surface or with pre-existing CKD
	Performance of e-alert system in unselected population of patients.
	Management of multiple alert in a same patient
	Influence of fluid balance/dilution [17].
Urine output	Difference in measurement according to setting (ICU vs. Ward, Specificity of paediatric units, rate of Foley catheter use).
	Management of missing data
	Errors in reading [8]
	Errors related to manual entry of urinary output
	Differences related to measurement (hourly vs. by shift vs. daily)
	Recognition of the lack of specificity of oliguria [18–20]
	Cross-tabulation between serum creatinine and UO

Systems to enhance recognition of AKI are promising strategies to improve the quality of care for AKI [9, 10]. Electronic medical records (EMRs) and clinical information systems (CIS) are becoming increasingly common in hospitals and can be leveraged to detect changes in serum creatinine or urine output according to current definitions for AKI. Such systems have the potential to increase AKI recognition, reduce the time to therapeutic interventions in order to prevent progression of AKI, and improve outcomes. Although there have been recent publications describing the implementation and evaluation of automated AKI alert systems, there has been no consensus on how such systems should be designed or implemented using EMRs and CISs, or whether refinements to the KDIGO AKI identification and staging system are required.

### Review

Our group recognized the need to develop principles for the design of automated AKI detection systems to produce real-time alerts using EMRs and CISs. We focused on how the current consensus criteria for AKI identification should be applied to this task, examined what refinements to the consensus criteria should be considered, and how AKI detection from such systems should be relayed to care providers. Our recommendations were framed by the notion that automated AKI alerts should be designed to enable early detection of AKI and provide opportunities to link AKI detection to clinical decision support tools for management, in order to mitigate avoidable propagation of AKI and associated harms.

#### ADQI process

We followed the ADQI process, as previously described [11]. The 15^th^ ADQI Consensus Conference Chairs assembled a diverse panel representing relevant disciplines (nephrology, critical care, pediatrics, pharmacy, epidemiology, health services research, biostatistics, bioinformatics and data analytics) from five countries in North America and Europe around the theme of “Acute Kidney Injury in the Era of Big Data” for a 2-day consensus conference in Banff, Canada on September 6–8, 2015. From this group, our work group was tasked with examining the application of EMRs and CISs for alerts for detection of AKI. Our pre-conference activities involved a systematic search of the literature for evidence on automated AKI alerts and a critical evaluation of the relevant literature. A pre-conference teleconference involving work group members was used to identify both the current state of AKI alerts and limitations in the current literature and clinical practices. The key questions for the work group were formulated from this discussion. During the conference our work group developed consensus positions, and plenary sessions involving all ADQI participants were used to present, debate, and refine these positions. Following the conference this final report was produced, revised, and approved by all members of the work group. The broad objective of ADQI is to provide expert-based statements and interpretation of current knowledge for use by clinicians according to professional judgment and identify evidence care gaps to establish research priorities.

#### Key questions

We identified 4 key questions, which were used to develop consensus statements:

1. What features of the current AKI consensus definitions should be applied to automated AKI alerts?
2. What relevant inputs could be used to refine automated AKI detection tools?
3. What are the key outputs from automated AKI detection systems which will be used to improve clinical responses and interventions?
4. What are the most important limitations and knowledge gaps regarding automated AKI detection that should be addressed through further research?

#### Overview of existing literature on automated AKI alert systems

A summary of publications reporting design, implementation, or evaluation of automated AKI alerts is provided in the Additional file 1: Table S1. We identified 12 automated AKI alert systems from 15 publications [12–29]. Eight of the systems were implemented in adult hospitals, one in a pediatric hospital, and 3 were specific to intensive care units. All systems included an AKI detection algorithm based on a change in serum creatinine, but only the 3 systems implemented within intensive care units also incorporated urine output criteria for AKI. Most systems aligned with consensus definitions for AKI detection and/or staging (RIFLE, AKIN, or KDIGO), while two systems used a non-consensus definition. The mode of delivery of alerts varied substantially and included interruptive as well as non-interruptive alerts delivered within EMR/CIS systems, by paper notification, paging systems, or telephone calls to providers. Five studies reported an evaluation of appropriateness of the AKI alert based on a reference standard (nephrologist or other adjudicator) and 4 studies examined the impact of the AKI alert on processes of care or clinical outcome.

#### What features of the current AKI consensus definitions should be applied to AKI alerts?

**Box 1:** **What features of the current AKI consensus definitions should be applied to AKI alerts?**

Consensus Statements: We agree with designing AKI alert systems to align with the existing KDIGO classification system, incorporating identification of baseline serum creatinine when known, changes in serum creatinine, and urine output when available. Basic AKI alert tools can be built using laboratory information systems and triggered by a single abnormal creatinine measurement, changes in inpatient creatinine measurements alone, or changes in inpatient and outpatient creatinine measurements. AKI alerts should be used as an opportunity to prompt earlier clinical evaluation, further testing and ultimately intervention, rather than as a diagnostic label.	

Several studies have assessed the feasibility and impact of electronic alert systems for AKI; however, surprisingly few of them evaluated the sensitivity and specificity of the AKI detection algorithms employed [12–30]. Where provided, results suggest a broad range in sensitivities and specificities for detecting AKI, which range from fair [25, 30] to excellent [12, 14] depending on the alert criteria and reference standard used for identification of true cases of AKI. Many of the studies that have reported on performance of automated AKI detection systems have been limited to a relatively specific spectrum of patients. The population of interest for large scale deployment of AKI detection systems may also differ substantially from the study populations examined to date because many of these studies have excluded patients with previously known chronic kidney disease (CKD) from these analyses despite their increased susceptibility to AKI. It is important to highlight that despite the application of published consensus criteria for AKI detection, the optimal algorithm for automated real time detection of AKI in clinical settings is currently unknown and comparison of potential algorithms is an important objective to be addressed by future studies.

Acknowledging the absence of evidence for optimal AKI alert criteria, our work group agreed that it was appropriate for initial AKI alert systems to align with the existing KDIGO definition and classification system for AKI (Table 1). Thus, algorithms should ideally identify a baseline serum creatinine when known, changes in serum creatinine, and urine output where feasible [31]. Urine output will be most accurately measured in patients using a Foley catheter and would require reporting in a 6 – 12 h time frame to align with consensus AKI definitions. Such a system would necessarily require electronic medical recording devices or frequent manual entry of data, which are vulnerable to errors in urine output measurement or reporting [32]. While this may be feasible in ICU settings, it is unlikely to be accurate or feasible in the majority of hospitalized patients where it would be impractical and inappropriate to prolong the placement of urinary catheters merely for the purpose of AKI detection.

**Fig. 1:**
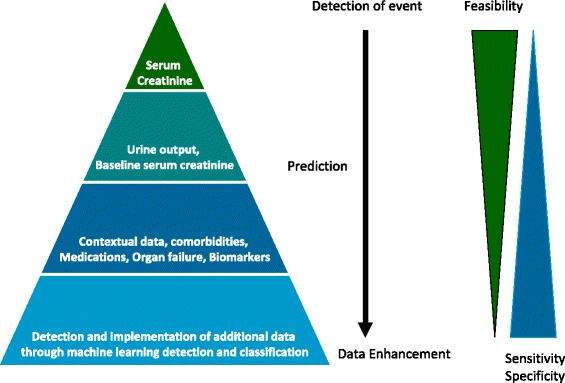
An approach to development and refinement of automated AKI detection systems. The scheme illustrates the potential to refine AKI alerts based on the current KDIGO criteria through the incorporation of additional data elements. Alerts based on serum creatinine are currently feasible in many EMRs / CISs; however, electronic data enhancements may improve the performance (sensitivity and specificity) of electronic alerts for AKI in the future. Reproduced with permission from ADQI

We believe that automated AKI alerts systems should be viewed as an opportunity to prompt clinical evaluation rather than provide a diagnostic label, and thus do not believe that urine output measurements are mandatory for basic AKI alert systems. At present, it is most feasible for hospital-based AKI alerts to be based on laboratory information systems and triggered by either a single abnormal creatinine measurement, significant creatinine elevations compared to estimated baseline serum creatinine, or by changes in serum creatinine measurements from an identified baseline in hospitalized patients [31]. Such a system would be imperfect and lack sensitivity with respectto the current KDIGO definition. The lack of urine output in such alert systems will likely fail to detect some episodes of AKI, detect AKI at a later time point, or under represent the severity of AKI in some patients [33]. The potential surrogates chosen to replace an unknown baseline creatinine also have several limits. Use of admission serum creatinine as the baseline will fail to detect AKI present at the time of admission while use of an estimated baseline may ignore pre-existing CKD leading to a high rate of false positive detection of AKI [33–35]. However, as both AKI and CKD are associated with adverse outcomes in hospitalized patients, and share some common principles in terms of management and medication safety, we do not think that concerns about misclassification of AKI and CKD should be a barrier to development of these systems. We encourage an incremental approach to development and modification of AKI alert systems with the potential to build in increasing complexity to allow alignment with AKI detection algorithms based on the KDIGO criteria (Fig. 1).

Several areas of uncertainty about AKI detection using the current KDIGO criteria remain (Table 2) [36–39]. Each of them is likely to modify the rate of false positive and negative alerts and should be addressed through future research to evaluate potential improvements in performance of automated AKI detection. In order to ensure the generalizability of these alerts to real-world settings, pragmatic cohorts in unselected populations of patients are needed. Ideally, future work in this field should characterize the rates of false positive and false negative alerts against a suitable clinical reference standard or measure of acceptability for physician, health care providers, and patients.

#### What relevant inputs could be used to refine AKI automated detection tools?

**Box 2:** ***What relevant inputs that could be used to refine AKI automated detection tools?***

Consensus Statements: Contextual information could be used to identify patients where AKI alerts are generated in order to increase their specificity or could be incorporated within alerts themselves to inform further diagnostic or treatment approaches. We suggest refining AKI alerts using readily accessible clinical, laboratory, and medication information for patients in whom AKI is detected to increase the appropriateness of AKI alerts and to link these alerts to actionable recommendations.	

Changes in creatinine concentration are frequently influenced by factors beyond changes in renal function. For example, volume of distribution, laboratory precision, presence of chromogens that interfere with measurement, and biological variation in creatinine production can contribute to false positive alerts as well as the failure to detect AKI [40–46]. Current definitions of AKI were developed to create a uniform definition to allow for comparison across clinical studies [47]. While the current definition facilitates epidemiological analysis and an improved understanding of associated outcomes, the performance of the current KDIGO definition for clinical case identification is unclear. Recent analyses suggest that the false positive rate for creatinine based alerts that are uninformed by other considerations may be as high as 30 % among certain population subsets [41].

False positive alerts are likely to be recognized as such by the clinicians receiving them, thereby mitigatingthe possibility of direct harm to patients. The true harm however of high false positive rates is “alert fatigue” and the risk of clinicians ignoring alerts even when accurate, thus rendering them ineffective. Furthermore, identification of a complex syndrome such as AKI, which can result from several causes and is frequently multifactorial, may not be useful to providers if the corrective action remains unclear. Providing AKI alerts within a specific clinical context provides increased opportunities to link alerts with a proposed meaningful response.

Strategies to lower the false positive rate of an alert will require the use of additional inputs beyond just changes in creatinine and/or urine output and should include patient risk factors, susceptibilities, and exposures. Incorporation of patient characteristics would allow for tailoring of AKI alert thresholds and may even create opportunities for identification of AKI in circumstances where it might have previously gone undetected. This would provide the potential to decrease the risk of false positives, while also providing the possibility of tailoring alerts to individual circumstances and providing recommendations for possible beneficial interventions.

Most current AKI alert systems rely on changes in serum creatinine determined within laboratory information systems. Given the current challenges of integrating real time physiological measurements, the adoption of additional data inputs has been slow and most efforts have instead focused on ensuring an accurate estimate of the change in creatinine [47]. Recognizing that the extent of adoption of EMRs and their current abilities are highly variable across health care systems and countries, we still believe that EMRs provide great potential to provide contextual information that could be used to inform AKI alerting systems. Potential sources of data to improve AKI alerting would include current medications, procedures, anthropometric measurements (rapid weight gain suggesting changes in volume of distribution), hemodynamic data (documentation of recent hypotensive episodes), time stamps (for events, procedures), comorbidities as well as historical clinical and administrative data. The use of natural language processing techniques, although still in their infancy in both development and implementation, provides the prospect of rapid search through physician and clinician documentation for data to incorporate into an alert system.

Increasing adoption of electronic order entry systems also provides an opportunity to improve AKI alerts. The identification of an extended exposure or the use of multiple concomitant agents resulting in an alert has been shown to be effective in lowering the incidence of AKI in pediatric populations [17]. Changes in anthropometric measurements could also be used to refine AKI alerts. Rapid weight increases that result from rapid volume expansion should result in a drop in creatinine if associated with stable renal function [46]. Correction of creatinine changes for fluid accumulation could be used to refine AKI alerts to increase their sensitivity before a change in creatinine occurs that would meet the traditional definitions for AKI. Additional information such as a history of AKI following prior exposure to contrast or a nephrotoxin on a prior admission may be retained in EMRs/CIS and used to trigger alerts within order entry system when the same or similar medication is prescribed. Information about such past episodes are typically not readily accessible from discharge summaries but would be available in historical datasets for patients and best identified using automated systems. Creatinine change is a late marker of AKI and novel biomarkers have the potential to identify the onset of kidney injury sooner. Should the development and clinical validation of any novel AKI biomarker lead to its widespread uptake, incorporation of the biomarker in any AKI alert system would be essential and could be of particular use in distinguishing true renal injury from changes in creatinine that are not accompanied by evidence of renal injury.

Big data approaches to identifying AKI will include the potential to use repeated laboratory measures while accounting for biological variability in measurements and the incorporation of large volumes of non-discrete data that would require both advanced detection and interpretation techniques. Continued refinement of the approach for detection of AKI will require the incorporation of both traditional parameters that we are aware of and nontraditional parameters that, while associated with AKI, need not be part of the causal pathway and may or may not directly inform the intervention that would allow patients to benefit from early recognition of AKI.

#### What are the key outputs from automated AKI detection systems which will be used to improve clinical responses and interventions?

**Box 3:** **What are the key outputs from automated AKI detection systems which will be used to improve clinical responses and interventions?**

Consensus Statements: Basic AKI detection systems should provide alerts to care providers as close to the time of AKI onset as possible. More sophisticated AKI alerts should identify the severity of AKI so that detection can be linked to graded responses and means of notifying care providers. AKI alerts could also be delivered when there is progression of AKI severity or when recovery is detected to allow surveillance, identify patients for research studies, and aid with resource planning based on the type of care needed.	

The output from automated AKI detection systems can be customized based on the capabilities of EMR/CIS systems and will need to be tailored to resources available locally. The most basic AKI alert system would be a passive display as part of an EMR or laboratory information system. Increasing complexity involves the ability to provide AKI alerts outside of these systems and should extend to linking alerts to communication systems beyond the system creating the alert. We believe that an ideal AKI alerting system would have the ability to modulate the delivery method of the alert based on its severity and need for clinical response. For example, it may be sufficient for an episode of stage 1 AKI to trigger the creation of a passive alert within the EMR that would only be triggered when the user is interacting with the EMR. The development of stage 2 AKI could trigger an alert that utilizes a hospital paging system or text messaging service directly to a specified clinician caring for the patient.

A second important output of an AKI alert system would provide a more active and interruptive alert during specific actions by clinicians. This form of an alert more closely ties AKI detection to recommendations provided in clinical decision support systems, such that the alert is brought to the attention of a user performing specific acts that may have a detrimental effect on renal function. An example would include an alert for AKI that is generated when a nephrotoxic medication is ordered. A simplified system may only alert the care provider to the presence of AKI as part of a medication ordering process. More advanced, predictive systems may be developed that trigger an alert whenever an intervention would be predicted to cause AKI or increase the stage of injury. The system could also make specific recommendations on medication or appropriate dose modifications [48, 49]. Within any EMR with a clinical decision support system it is important that human factors are considered to develop strategies to prevent or reduce alert fatigue. Modulating the intrusiveness of an AKI alert depending on AKI severity or on the likelihood that an action may cause injury will increase the effectiveness of the alert, prevent alert fatigue, and ensure an appropriate response.

A third feature of output from an automated AKI alert system could include a message to a registry system to permit tracking of specific AKI quality indicators and resource planning. Such a system would also facilitate research by providing information on the presence and timing of development of AKI in patients, collection of factors contributing to the alert trigger, and monitoring for subsequent interventions by the clinicians and eventual outcomes. AKI surveillance based on AKI alert outputs could also be used to plan for resource allocation (e.g. need for staff and equipment for dialysis) and identify patients for enrollment in prospective research studies.

#### What are the most important limitations and knowledge gaps regarding automated AKI detection that should be addressed through further research?

Understanding the limitations and knowledge gaps regarding existing automated AKI alert applications is important to spur further research and innovation. The developers of automated AKI alert systems will need to confront and overcome these current limitations, but more importantly will need to focus on several novel areas of advancement. Ideally AKI alert systems will not just detect first onset of AKI but will continuously and automatically monitor and assess a patient’s risk for developing AKI. To do so they will need to integrate in real time the wealth of clinical data available for a patient and assess both static and dynamic data elements of a patient. Advanced AKI alert systems will need to leverage the information that is available or will soon be available from systems such as continuously reporting sensors which are either worn by the patient or placed in their proximity. Perhaps the next generation of AKI alert systems will enable a move beyond binary detection (AKI yes or no) or categorical output (AKI stage), to provide a continuous score or dashboard presentation of AKI that encompasses both AKI severity, rate of progression, and other features of the clinical context. Assessing the performance of new AKI alert systems will require measuring both the diagnostic capability of a system and its performance within the larger data acquisition and processing system. The acceptability of an alert system to healthcare providers, patients and administrators will also need to address secondary issues such as the utility of AKI alert systems in research and surveillance. The implications of AKI alert systems for medical liability will also need to be defined. Finally, as both the sophistication and performance of AKI alert systems improves, the possibility of using data mining techniques and predictive analytics to discover new associations within clinical data that better detect or even predict AKI will become real.

## Conclusion

In this review we have articulated some principles for developing automated, real time AKI alert systems within EMRs / CISs. We encourage alignment and evaluation of modifications to the most recent consensus definitions and classification schemes for AKI, with the understanding that an effective AKI alert system must rely upon data that can be made available within the EMR or CIS. Currently available applications for the detection of AKI using EMRs and CISs are in their infancy. Given the prevalence of AKI, the morbidity and mortality associated with even mild and moderate degrees of AKI, and the silent nature of the condition, the importance of developing better tools to detect cannot be overstated. We hope the consensus statements developed in this review can help provide a roadmap for future development.

## Additional file

Additional file 1: Table S1. Characteristics of existing AKI alert systems. (DOC 50 kb)

## Abbreviations

AKI: acute kidney injury
EMR: electronic medical record
CIS: clinical information system
CKD: chronic kidney disease
SCr: serum creatinine
