# Supplementary material for: Applications for detection of acute kidney injury using electronic medical records and clinical information systems: workgroup statements from the 15th ADQI Consensus Conference
Source: Can J Kidney Health Dis. 2016 Feb 26;3:9. doi: 10.1186/s40697-016-0100-2 (PMC4768328; doi:10.1186/s40697-016-0100-2)
Supplement: Additional file 1: Table S1. — Characteristics of existing AKI alert systems. (DOC 50 kb) [file 40697_2016_100_MOESM1_ESM.doc]

Supplementary Table: Characteristics of existing AKI alert systems

| **Author,**  **year** | **Setting** | **Population eligible to receive alert** | **AKI criteria used for alert** | **Delivery of alert** | **Additional features accompanying alert** | **Implementation Activities** |
| --- | --- | --- | --- | --- | --- | --- |
| Colpaert, 2007, 2012 | Surgical and medical icu in tertiary care hospital; Ghent University Hospital, Belgium | All patients in surgical and medical ICU; excluded if <18 years, history of CKD stage 5 (chronic renal replacement or GFR<15mL/min), renal transplantation within 3 months, nephrectomy or recent kidney trauma, admission episode overlapping both phases of the study, or technical problems (server problem, ICES connection lost, AKI sniffer software bug) | Based on RIFLE criteria; collects data from commercial central care information system (ICIS) that monitors, pumps and lab data; based on urine output and serum creatinine | Synchronous interruptive alert (if not acknowledged, alerted again in 10 minutes), Sent to intensivists as an electronic alert using Digital Enhanced Cordless Technology (DECT) telephone (similar in appearance to cell phone/pager) | Included RIFLE class and criterion responsible for alert (urine vs. serum creatinine) | Residents received education about evaluation and therapeutic intervention for AKI prior to implementation of alert. |
| McCoy, 2010, 2012 | Vanderbilt University Hospital, Nashville, TN, academic tertiary-care facility | Adult patients experiencing increasing creatinine levels, prescribed medications to be avoided and with baseline creatinine clearance scr >30mL/min , excluded dialysis before first serum creatinine measure, died transferred to external facility, or discharged immediately | minimum 0.5mg/dL increase in scr over 48 hours after order of nephrotoxic or renally cleared medications | Passive noninteractive warning appears on computerized provider order entry interface and on printed rounding reports; for contraindicated or high toxicity medications, or when provider had not adjusted medication based on passive alert an interruptive alert asked providers to modify/discontinue target orders, or mark current dosage as correct, or defer alert on exiting order entry system | Integration with medical order systems, provided dosage advice, assistance with dosage; includes graph of recent serum creatinine changes and recent urine output, recommendations about which medications should be discontinued, dose adjusted or considered for changes; |  |
| Garzoto, 2011 | Ten ICUs in Italy | Excluded patients with end-stage renal disease | Based on RIFLE criteria-both creatinine and UP criteria: >50% SCr increase from baseline or reduction in UO to <0.5mL/kg/h for >6 hours | Data collected via user-friendly web-based data collection tool and an optional alert can be activated when patient reaches RIFLE criteria; pop-up alarm blinks and operator (the person inputting measures) must decide for a RIFLE-based application of RRT technique | Includes RIFLE class, baseline creatinine and actual creatinine (multiple of baseline) | N/A |
| Thomas, 2011 | 2 separate hospitals in Birmingham, England | Referred patients Ages 16 and older, excluded those referred for dialysis at time of first alert, and outpatients | Triggered by >=75% rise in creatinine from previous value; staging using RIFLE or AKIN stage (uses rise from baseline to peak) | Sent by integrated clinical environment pathology system |  |  |
| Selby, 2012 | Royal Derby Hospital | Patients at hospital excluding those in renal unit | AKIN criteria based on serum creatinine measures, 50% over baseline flagged; estimated baseline on MDRD equation; pathology computer system compares all measured creatinine; baseline from within 12 months prior (rather than 48 hours-lowest creatinine used) | Real-time electronic reporting system, results reviewed by clinical chemist, report issues to hospital results reporting system; daily electronic report of all AKI episodes automatically generated | Linked to AKI clinical guidelines, and AKIN diagnostic criteria | Combined with cross-discipline education for medical and nursing staff, publication of easily available clinical guidelines on hospital intranet that are linked to reporting system and improved mechanism for Nephrology referral |
| Goldstein, 2013, Kirkendall, 2014 | Quaternary pediatric hospital Medical and Surigical Service Chiefs at Cincinnati Children’s Hospital Medical Centre | Non-critically ill hospitalized children receiving intravenous aminoglycoside for >=3 days or >=3 nephrotoxins simultaneously ; excluded children in ICU, chronic kidney disease, kidney transplant or urinary tract infection | Modified pediatric RIFLE criteria (>=decrease in estimated scr clearance); data collected through electronic hospital records; daily scr monitoring | HER trigger warning; Automated trigger report sends information to rounding pharmacists; was initially a manual trigger but converted into electronic triggers; trigger reports send to pharmacists in email embedded with URL links | Recommends substitution of nonnephrotoxic or less nephrotoxic medication or pharmacokinetic drug concentration monitoring; Identified medications that put patient at risk of nephrotoxicity | Adherence to recommendations reported to PI; |
| Ahmed et al., abstract 2014, paper 2015 | derivation cohort: Olmstead County, MN, residents admitted to all Mayo ICU tertiary care centers  Validation cohort: Mayo clinic, Rochester campus medical/surgical ICU | Age ≥18 years and previously gave permission for research using EHRs. Excluded patients with evidence of AKI at admission. | Serum creatinine level and hourly urine output based on actual body weight; based on AKIN criteria | Synchronous (real time) with up to 1 hour delay; screens patient records at 15 minute intervals; does not detail who receives alert or how | N/A | N/A |
| AKI NHS Patient Safety Alert (Think Kidneys); 2014, | All hospitals covered by NHS (England) | Renal units excluded to prevent patients with ESRD being included and neonatal units excluded; primary care locations (future roll-outs planned) | based on NHS England patient safety alert; if ratio of reference scr/recent serum creatinine measure is >1.5 and patient >=18 will send a report; if patient <18 or the change occurred within 48 hours or the difference is >26umol/L then alert will be sent AKIN stages 1-3 reported | Automated computer software program algorithm as part of laboratory software; results/alert must be communicated to clinicians in a communication system developed locally based on current resources; can be reported like other results or can trigger alert | Alerting system to be developed locally; at minimum, results must be sent to hospital reporting systems; may be laboratory staff telephone AKI warning stage to clinicians (high volume of work), could be within patient management systems, via dedicated messaging platforms or through electronic ‘track and trigger’ patient observation systems | This is an attempt to standardize AKI detection across England; program plans for audit and feedback in future; Data extracted and sent to UK renal registry to facilitate quality improvement and benchmarking; recommends education prior to roll-out and ongoing education programmes, introduction of AKI guidelines, AKI care bundles |
| Porter, 2014 | Nottingham University Hospitals, UK; Large National Service Hospital | >16 years and no chronic dialysis; | RIFLE and AKIN; serum creatinine rising; stage 1 alert-50% increase in SCr within 7 days or 0.3mg/dL within 48 hours; where RIFLE and AKIN differ in terms of AKI stage, higher stage is reported; if no baseline ‘theoretical scr’ calculated using MDRD equation assuming normal GFR | Synchronous Real-time automated electronic alert (e-alert) system presented to clinician; passive alert non-interruptive; issued to clinician accompanying the qualifying scr result | Includes AKI stage and referral to intranet-based AKI guideline for management |  |
| Flynn, 2015 | Laboratory at University College London Hospitals NHS Foundation Trust (UCLH) | All patients served by laboratory (inpatients, outpatients, primary care) | 50% increase in creatinine to a concentration >50umol/L within 90 day period | Synchronous Real-time automated alert; creatinine >100umol/L or from patients <18 years received increase alert prominence in electronic records and are phoned to requesting physician as part of laboratory procedures for phoning critical results; list of all alerts emailed to intensive treatment unit (ITU) outreach twice a day via automated scheduled query all creatinine results >300umol/L reviewed twice daily by biochemist and phoned if AKI suspect | Link to local AKI guidelines; | Information material provided (AKI guidelines) |
| Sawhney, 2015 | Grampian region of Scotland | Patients with renal impairment, a case-note review by nephrologist using RIFLE to diagnose AKI and CKD; excluded patients living outside Grampian, patients on RRT, patients with no case notes and patients without a community health index number | Used algorithm that the NHS England AKI developed (above) | Changes in scr tracked in biochemistry system; no real time alerts given out in the study. Intent was to compare AKI diagnosis between the NHS algorithm and the clinician diagnosis |  |  |
| Wilson, 2015 | University of Pennsylvania, PA, USA; tertiary care hospital | Age ≥18 years Excluded patients with initial hospital creatinine 4.0mg/dL or greater, fewer than 2 creatinine measures, unable to determine coverage provider, admission to hospice/observation unit, , end-stage renal disease. | Acute kidney injury defined by the Kidney Disease Improving Global Outcomes creatinine-based criteria: scr<0.3mg/dL greater than value from previous 48 hours or 50% greater than previous 7 day value; included outpatient scr values; changes in scr in real time; | Synchronous (real time) with up to 1 hour delay (alerts sent in hourly batches); covering provider (intern, resident, nurse practitioner) and unit pharmacist receive text message on hospital-provided cell phone alerting them of AKI (utilizes existing paging technology used in the hospital); 56% used wireless communication protocol with automatic confirmed receipt and others sent via simple mail transfer protocol; | Text includes link to website with link to KDIGO AKI guidelines, told provider to take appropriate diagnostic and therapeutic measures | Website included information about the study |
